# Fast intermolecular interaction energy calculation with the OPLS-AA force field

**DOI:** 10.1186/s13321-026-01264-9

**Published:** 2026-08-06

**Authors:** Mirco Daniel, Hannah Kullik, Martin Urban, Christoph Steinbeck, Achim Zielesny

**Affiliations:** 1 Institute for Bioinformatics and Chemoinformatics, Westphalian University of Applied Sciences, August-Schmidt-Ring 10, 45665 Recklinghausen, Germany; 2 Microverse Center Jena, Friedrich-Schiller University, Rosalind Franklin Strasse 5, 07745 Jena, Germany

**Keywords:** Intermolecular energy, Monomer, Dimer, Molecular force-field, OPLS-AA, LigParGen, BOSS

## Abstract

A fast pipeline based on the OPLS-AA force field is presented that enables the automated calculation of non-bonding intermolecular (dimer) interaction energies derived from a set of small organic (monomer) molecules. To calculate the non-bonding contributions, optimized geometries of the monomer molecules as well as their OPLS-AA van der Waals and atomic partial charge parameters are required. The key advantage of the new pipeline lies in its fast, highly parallelized in-memory computations without slow I/O operations, which make it possible to thoroughly process comparatively large numbers of (more than a hundred) monomer molecules within acceptable time frames (hours and days): Compared to an analogous, more versatile, and comprehensive approach, the new computational scheme delivers comparable results while being more than two orders of magnitude faster. For locally estimating the required OPLS-AA force field parameters with the LigParGen and BOSS software packages, a user-friendly graphical user interface for the Windows operating system is provided.

**Scientific contribution**

Rapid calculation of mutual intermolecular energies for a set of monomer molecules based on the widely used OPLS-AA force field enables a considerable expansion of this type of calculation for practical purposes. The performance improvement can be used to achieve significant improvements of interaction energy averages as well as considerable expansions in the size of the monomer molecule set.

## Introduction

The quantitative estimation of intermolecular interactions between (small organic) molecules substantially supports both their theoretical understanding and practical design purposes, from materials science to life sciences [1, 2]. Compared to the total energy of a configuration of a molecular dimer (i.e., a non-covalently bound pair of monomer molecules) or the covalent bonds within its constituent monomers, the contribution of intermolecular non-bonding interactions is typically orders of magnitude smaller, as it only includes dispersed variations of electromagnetic interactions due to van der Waals forces or partial atomic charges.

A recent work presented an automated molecular force-field based calculation pipeline for the estimation of mutual intermolecular interaction energies of a set of small to medium-sized organic monomer molecules [3], openly available on GitHub [4]. The calculation pipeline consists of a computing backbone programmed in Java, with all force-field-based calculations being performed by stand-alone, task-based executables of the Tinker Molecular Modelling package [5]. This architecture relies heavily on comparatively slow file I/O operations for communication between the different executables. This creates a severe performance bottleneck when extending the workflow to larger monomer sets, as the computational effort scales quadratically with the number of monomers.

The aim of this work is to introduce an algorithmically simplified variant of this calculation pipeline specifically for the OPLS-AA (Optimized Potentials for Liquid Simulations–All Atom) force field [6, 7], where the final dimer geometry optimization is skipped. The new variant is again highly parallelized and thoroughly exploits modern multi-core workstation processors. Unlike the established pipeline, however, it performs all calculations exclusively in memory within the Java backbone, without invoking an external Tinker executable file, thereby completely avoiding comparatively slow communication-based I/O operations. For obtaining the OPLS-AA force field parameters of the organic monomer molecules, the LigParGen calculation engine (either locally or via a webserver) can be utilized [8].

For local LigParGen calculations with the Windows operating system, a supplementary, standalone open graphical user interface (GUI) application based on a local executable of the BOSS software [9] is provided, that alleviates parameter calculation for large sets of monomer molecules. A convenient Windows installer is available in addition.

## Methods

### Intermolecular energy related definitions

The force-field-based intermolecular energy ${E}_{ij}^{C}\left(r\right)$ between two monomer molecules i and j depends on the intermolecular distance r between the centers of the molecules as well as their relative spatial configuration C. For a specific distance ${r}_{fix}$ there is a minimum energy configuration ${C}_{min}$ with ${E}_{ij}^{{C}_{min}}\left({r}_{fix}\right)\le {E}_{ij}^{C}\left({r}_{fix}\right)$ which is smaller than all other configuration energies ${E}_{ij}^{C}\left({r}_{fix}\right)$ at that distance. An averaged intermolecular energy at a fixed intermolecular distance ${r}_{fix}$ can be obtained by approximating the infinite number of possible spatial dimer configurations with a subset of configurations according to an elaborated scheme developed in [3], where configuration sampling at each fixed distance ${r}_{fix}$ involves evaluating ${N}_{sphere}\times {N}_{sphere}\times {N}_{rot}$ distinct spatial configurations. First, ${N}_{sphere}$ evenly spaced points are generated on a unit sphere around each monomer's center using a Fibonacci lattice. The molecules are aligned along an axis connecting their centers and one surface point from each sphere, creating ${N}_{sphere}\times {N}_{sphere}$ orientations. The second molecule is then rotated around this axis by ${N}_{rot}$ different angles. These ${N}_{sphere}\times {N}_{sphere}\times {N}_{rot}$ configurations can be averaged with Boltzmann weights ${w}_{C}\left({r}_{fix}\right)$ for a defined temperature (with ${\mathrm{k}}_{\mathrm{B}}$, the Boltzmann constant, and $\mathrm{T}$, thermodynamic temperature)

$$w_{C} (r_{fix} ) =_{e} - \frac{{E_{ij}^{C} (r_{fix} )}}{{K_{B} T}}$$

$$\langle E_{ij} \rangle (r_{fix} ,T) = \frac{{\sum_{C}^{{N_{Sphere} \times N_{Sphere} \times N_{rot} }} E_{ij}^{C} (r_{fix} )w_{C} (r_{fix} )}}{{\sum_{C}^{{N_{Sphere} \times N_{Sphere} \times N_{rot} }} w_{C} (r_{fix} )}}$$

to obtain an averaged intermolecular energy $\langle {E}_{ij}\rangle \left({r}_{fix},T\right)$ for the fixed distance ${r}_{fix}$. Among these ${N}_{sphere}\times {N}_{sphere}\times {N}_{rot}$ averaged configurations, the one with the minimal intermolecular energy is denoted ${E}_{ij}^{{C}^{*}}\left({r}_{fix}\right)$. Note that ${E}_{ij}^{{C}^{*}}\left({r}_{fix}\right)\ge {E}_{ij}^{{C}_{min}}\left({r}_{fix}\right)$, because the sampled configuration ${E}_{ij}^{{C}^{*}}\left({r}_{fix}\right)$ may only be an approximation–though usually an acceptable one–rather than the true minimum energy configuration ${E}_{ij}^{{C}_{min}}\left({r}_{fix}\right)$ at the fixed distance ${r}_{fix}$. The global minimum energy dimer is characterized by a distinct distance ${r}_{min}$ so that ${E}_{ij}^{min}={E}_{ij}^{{C}_{min}}\left({r}_{min}\right)\le {E}_{ij}^{C}\left(r\right)$, i.e., ${r}_{min}$ is the distance between the centers of two molecules i and j when the dimer geometry corresponds to the global energy minimum. The corresponding averaged minimum energy distance ${r}_{\langle E\rangle ,min}$ with ${\langle {E}_{ij}\rangle }^{min}\left(T\right)=\langle {E}_{ij}\rangle \left({r}_{\langle E\rangle ,min},T\right)\le \langle {E}_{ij}\rangle \left(r,T\right)$ does not necessarily coincide with minimum distance ${r}_{min}$ of the global minimum energy dimer but will generally be located in its vicinity.

The OPLS-AA force field estimates the intermolecular interaction energy ${E}_{ij}^{C}$ between two monomer molecules i and j as a sum of an electrostatic Coulomb $\left({E}_{ij}^{Coulomb}\right)$ and a Lennard–Jones 6–12 $\left({E}_{ij}^{LJ}\right)$ contribution [6]

$$E_{ij}^{C} = E_{ij}^{Coulomb} + E_{ij}^{LJ}$$

with

$$E_{ij}^{Coulomb} = \mathop \sum \limits_{a}^{Molecular\,i} \mathop \sum \limits_{b}^{Molecule\,j} k_{e} N_{A} f_{cal} \frac{{q_{a} q_{b} e^{2} }}{{r_{ab} }}$$

$$E_{ij}^{LJ} = \mathop \sum \limits_{a}^{Molecular\,i} \mathop \sum \limits_{b}^{Molecular\,j} \,4\varepsilon_{ab} \left[ {\left( {\frac{{\sigma_{ab} }}{{r_{ab} }}} \right)^{12} - \left( {\frac{{\sigma_{ab} }}{{r_{ab} }}} \right)^{6} } \right]$$

${k}_{e}$, Coulomb's constant $\frac{1}{4\pi {\varepsilon}_{0} } \left[\frac{N{m}^{2}}{{C}^{2}}\right]$; ${N}_{A}$, Avogadro's constant $\left[\frac{1}{mol}\right]$; ${f}_{cal}$, conversion factor from Joule to Calorie $\left[\frac{cal}{J}\right]$; e, elementary charge $\left[C\right]$; ${q}_{a}e$, partial charge of atom a in molecule $i; {q}_{b}e$*,* partial charge of atom b in molecule j; ${\varepsilon}_{0}$, vacuum permittivity $\left[\frac{{C}^{2}}{N{m}^{2}}\right]$; ${r}_{ab}$, distance between atom a in molecule i and atom b in molecule j $\left[m\right]$; ${\varepsilon}_{ab}$, depth of the potential well between atom a in molecule i and atom b in molecule j $\left[\frac{kcal}{mol}\right]$; ${\sigma}_{ab}$, distance at which the potential energy between atom a in molecule i and atom b in molecule j is zero $\left[\mathrm{m}\right]$.

The parameters ${\varepsilon}_{ab}$ and ${\sigma}_{ab}$ for pairs with different atoms are obtained with the (geometric mean) combination rules of the self-interaction parameters:

$$\varepsilon_{ab} = \sqrt {\varepsilon_{aa} \varepsilon_{bb} }$$

$$\sigma_{ab} = \sqrt {\sigma_{aa} \sigma_{bb} }$$

### Simplified calculation pipeline

The new simplified variant of the intermolecular energy calculation pipeline starts with optimized geometries of all monomer molecules and their corresponding OPLS-AA (q, ε and σ) force field parameters. The optimized molecule geometries may be obtained by a priori quantum chemical or molecular mechanics calculations to result in a set of monomer PDB-files, each containing the 3D coordinates of its monomer atoms. The OPLS-AA force field parameters q, ε and σ of each monomer molecule for the non-bonding contributions are taken from publicly available parameter sets (e.g., [10]) or obtained with the LigParGen calculation engine. LigParGen calculations are available via the new local LigParGen-GUI application (see below), the direct use of the native Linux LigParGen CLI or the LigParGen web server, which all take a monomer PDB-file as an input and generate the corresponding monomer xyz- (3D coordinate) and key-(parameter)-files for the calculation pipeline. The set of monomers xyz- and key-files is then copied to the input directories */Molecules* and */tinker/params/OPLSAALIGPARGEN* to calculate the mutual interaction energies.

**Fig. 1:**
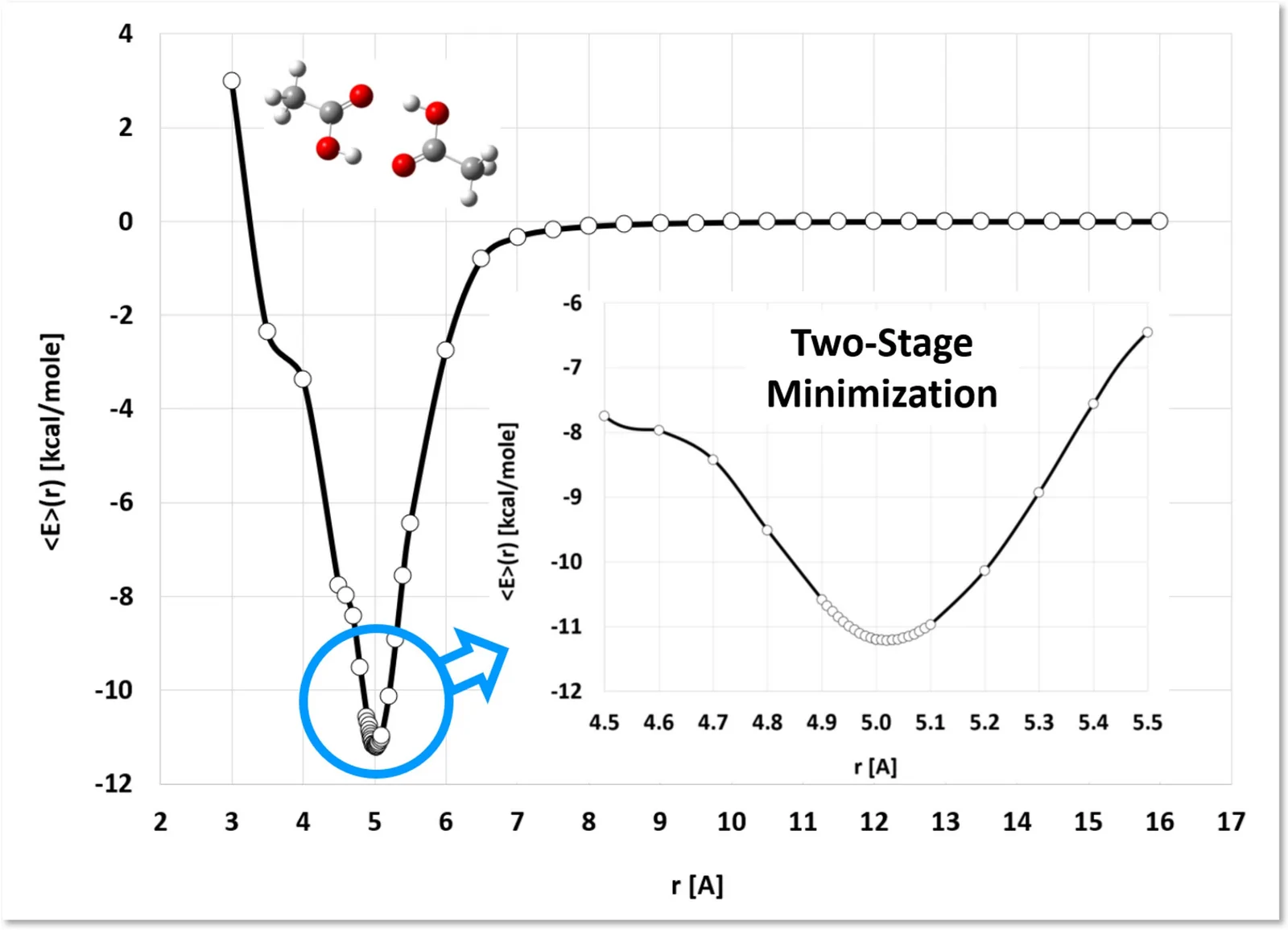
Estimation of ${\mathrm{r}}_{\langle \mathrm{E}\rangle ,\mathrm{m}\mathrm{i}\mathrm{n}}$ and corresponding ${\langle {\mathrm{E}}_{\mathrm{i}\mathrm{j}}\rangle }^{\mathrm{m}\mathrm{i}\mathrm{n}}\left(\mathrm{T}=298\mathrm{K}\right)$ for the acetic acid (HAc) dimer with an initial scan of intermolecular distances in the range from 3 to 16 ${\AA }$, in steps of 0.5 ${\AA }$. For each distance, $144\times 144\times 16$ dimer configurations are calculated according to the scheme in [3] to determine each averaged $\langle {\mathrm{E}}_{\mathrm{i}\mathrm{j}}\rangle \left(\mathrm{r},298\mathrm{K}\right)$ value. The determined global averaged energy minimum at about r = 5 ${\AA }$ is refined using a two-stage minimization procedure (with initial step size of 0.1 ${\AA }$ and 0.01 ${\AA }$ in the second refinement stage). The determined approximation to the global minimum energy dimer configuration is shown at the top

A typical estimation procedure for averaged interaction energies is sketched in Fig. 1 for the acetic acid (HAc) dimer: To estimate ${r}_{\langle E\rangle ,min}$ and corresponding ${\langle {E}_{ij}\rangle }^{min}$, an initial scan is performed with various intermolecular distances (typically in the range from about 3 to about 16 ${\AA }$, in steps of 0.5 ${\AA }$). For each distance, ${N}_{sphere}\times {N}_{sphere}\times {N}_{rot}$ dimer configurations are calculated according to the scheme in [3] to determine each averaged $\langle {E}_{ij}\rangle \left(r\right)$ value. The determined global averaged energy minimum is then refined using a two-stage minimization procedure (where the step size is initially set to 0.1 ${\AA }$ and subsequently to 0.01 ${\AA }$ in the second refinement stage) to obtain an approximate value for ${\langle {E}_{ij}\rangle }^{min}=\langle {E}_{ij}\rangle \left({r}_{\langle E\rangle ,min}\right)$ with an accuracy of two decimal places (0.01 ${\AA }$) for ${r}_{\langle E\rangle ,min}$. If desired, ${\langle {E}_{ij}\rangle }^{min}=\langle {E}_{ij}\rangle \left({r}_{\langle E\rangle ,min}\right)$ can finally be approximated by an increased number of ${N}_{sphere}\times {N}_{sphere}\times {N}_{rot}$ dimer configurations.

For the detailed settings of the calculation process there are two text files available: In the *MIPET.job* text file, the OPLS-AA force field and the monomer molecules for mutual interaction calculation have to be specified. In the *MIPET.properties* text file, additional parameters related to the pipeline's execution can be set. Most of the settings are self-explanatory and described within the text file itself. The default parameters are based on extensive tests and do usually not need to be modified. Crucial parameter settings are the number of dimer configurations to be calculated for each distance (*MIPETSphereNodeNumber* ${N}_{sphere}$, *MIPETRotationNumber* ${N}_{rot}$) and the number of CPU cores to be used in parallel (*MIPETCPUCoreNumber*), which determine the calculation speed. The calculation pipeline evaluates all dimer interaction energies according to the defined settings along the defined calculation scheme, and creates a subdirectory for each dimer interaction in its specified output path that contains all dimer-specific calculation results. These comprise general information about the intermolecular energies, intermolecular energy-distance plots, and the lowest-energy dimer configuration in PDB, xyz and tinker’s xyz format. The results may be further processed to obtain differential pair interaction energies or repulsion parameters for mesoscopic dissipative particle dynamics (DPD) simulations. For the latter, each result directory already contains a text file with particle set data for the open *MFsim*/*Jdpd* simulation environment [24–27]. The new variant of the calculation pipeline is openly available on GitHub [4].

### Local LigParGen-GUI application

**Fig. 2:**
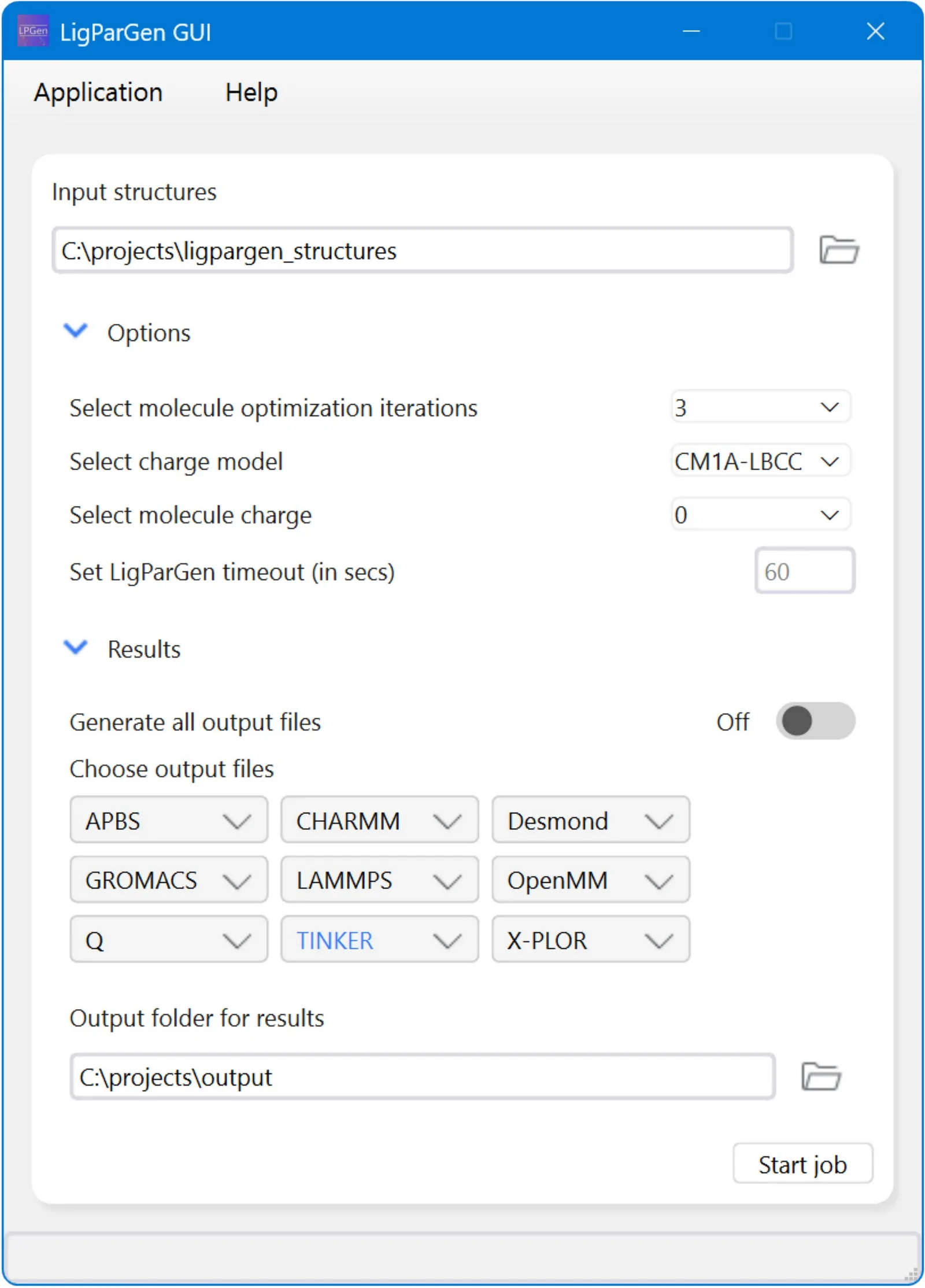
Graphical user interface of the local LigParGen Windows application

A new local LigParGen-GUI application is provided (see Fig. 2) as a supplementary, standalone tool that is not required for the calculation pipeline. It is currently only available for Windows 10 or later versions and facilitates the usage of the LigParGen v2.1 [11–13] and BOSS [14, 15] software packages, where the BOSS software package can be requested as a compressed archive file via the William L. Jorgensen laboratory website [9]. The application is implemented in Python 3.11 [16] using the PyQt6 framework [17] and follows a Model-View-Controller (MVC) architecture. Since BOSS is compiled against 32-bit Linux libraries, it requires a 32-bit Linux system. Thus, calculations are executed within the Windows Subsystem for Linux 2 (WSL2), while the GUI itself runs natively on Windows. This architecture was specifically chosen to lower the entry barrier for Windows users, who would otherwise face a notoriously difficult manual setup process or require dedicated Linux hardware to run BOSS. The LigParGen-GUI exposes the same configuration options as the LigParGen web server [12, 13, 18], including settings for the number of molecular optimization iterations, the charge model, and the molecule charge. It supports all output formats available through the LigParGen command-line interface (CLI) and adds a built-in timeout mechanism to terminate calculations exceeding user-defined time limits. Communication between the Windows host and the WSL2 environment is managed by a microservice that uses ZeroMQ [19] in a push/pull messaging pattern. Since the GUI functions as a mere wrapper around the native LigParGen CLI, advanced Linux users can of course completely bypass the Windows GUI and feed native CLI outputs directly into the calculation pipeline.

A typical workflow with the local LigParGen-GUI application begins with specifying an input directory containing either the molecules’ PDB-files or a text file with their SMILES strings. The user then configures the LigParGen settings and selects the desired output file formats before choosing an output directory for storing calculation results transferred from WSL2. Once the job is submitted, the GUI transfers the job to the WSL2 microservice which invokes the LigParGen CLI with the appropriate arguments and communicates status updates back to the GUI. Progress is displayed in a dynamically updated dialog window, implemented with PyQt6’s signal-slot mechanism to ensure thread safety. Upon completion, the software notifies the user and offers the option to automatically open the output directory.

The local LigParGen calculations require the BOSS software package as a compressed archive file which can be installed directly through the LigParGen-GUI user interface: At first launch, the application prompts the user to provide the path to the BOSS ZIP-file.

The graphical user interface is distributed as a standalone executable generated with PyInstaller [20]. It includes a custom AlmaLinux distribution [21] that is automatically imported into WSL2 during installation and provides a preconfigured LigParGen environment. A convenient Windows installer executable built with Inno Setup 6 [22] is also provided. Documentation is available in PDF format through the Help menu. The complete LigParGen-GUI source code is openly accessible on GitHub [23].

## Results and discussion

**Fig. 3:**
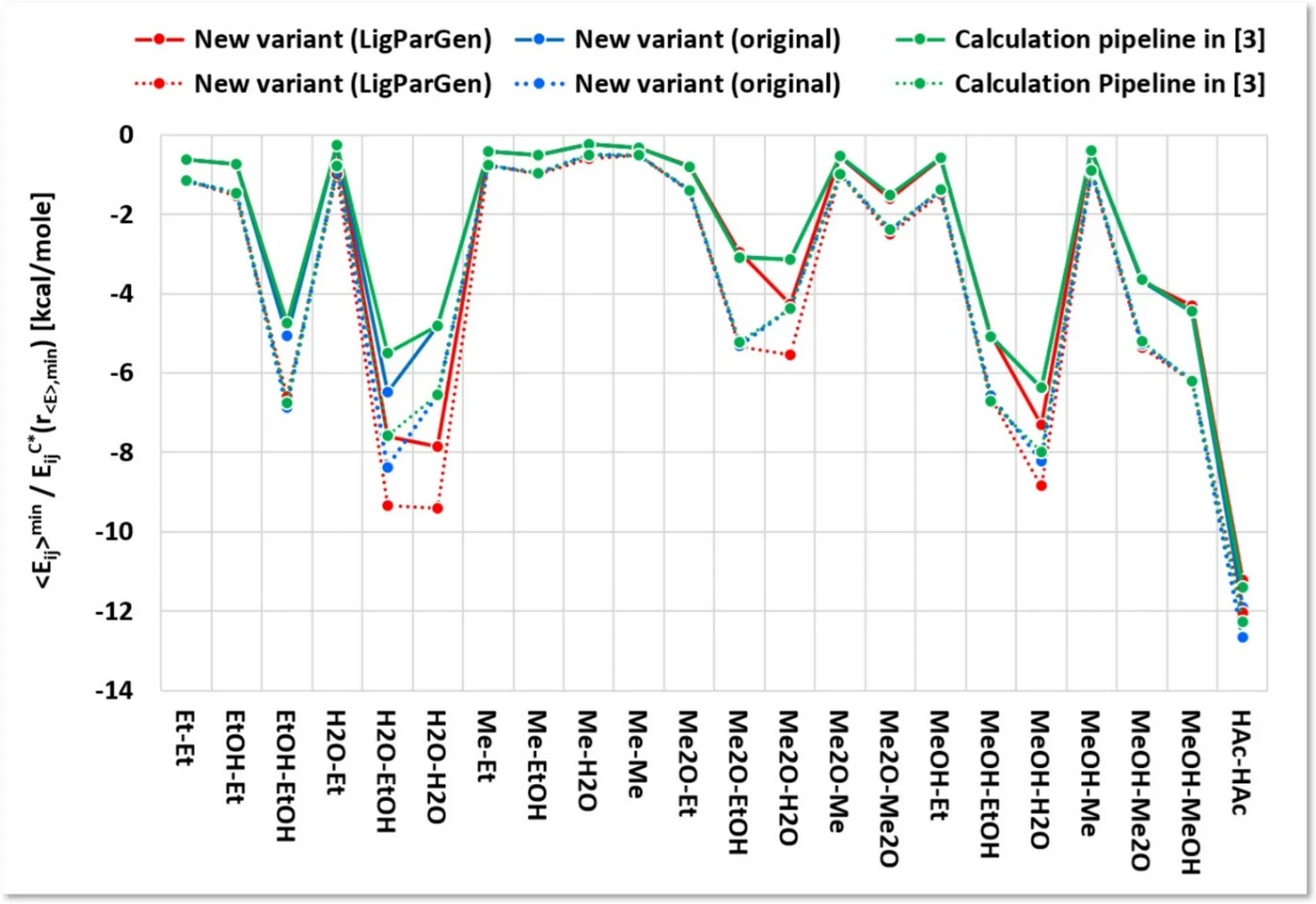
OPLS-AA intermolecular interaction energies (with the TIP5P model for water) in kcal/mole for different dimers and calculation pipelines at the corresponding minimum energy distances of the monomer molecules (see Table 1). The solid lines refer to ${\langle {\mathrm{E}}_{\mathrm{i}\mathrm{j}}\rangle }^{\mathrm{m}\mathrm{i}\mathrm{n}}$, the dotted lines to ${\mathrm{E}}_{\mathrm{i}\mathrm{j}}^{{\mathrm{C}}^{*}}\left({\mathrm{r}}_{\langle \mathrm{E}\rangle ,\mathrm{m}\mathrm{i}\mathrm{n}}\right)$

The calculation results of the new pipeline variant can be directly compared to those of the dimer calculations provided in [3]: The analog Table 1 and corresponding Fig. 3 demonstrate that the new pipeline is on par with the established more complex approach. Since there is no final geometry optimization of the dimer, the new pipeline cannot calculate ${E}_{ij}^{min}$, but the results for the approximations ${E}_{ij}^{{C}^{*}}\left({r}_{\langle E\rangle ,min}\right)$ and the averages ${\langle {E}_{ij}\rangle }^{min}$ are in good agreement and close to ${E}_{ij}^{min}$. The use of LigParGen generated parameters also leads to comparable results to those of the original OPLS-AA force field (where original charges of TIP5P water are multiplied by a factor of 1.14, as is the case in the LigParGen calculations).

**Table 1:** OPLS-AA intermolecular interaction energies (with the TIP5P model for water) in kcal/mole for different dimers and calculation pipelines at the corresponding minimum energy distances of the monomer molecules (averages are obtained at $\mathrm{T}=298 \mathrm{K}$)

	New variant (LigParGen)		New variant (original)		Calculation Pipeline in [3]		
Dimer	${\langle {\mathrm{E}}_{\mathrm{i}\mathrm{j}}\rangle }^{\mathrm{m}\mathrm{i}\mathrm{n}}$	${\mathrm{E}}_{\mathrm{i}\mathrm{j}}^{{\mathrm{C}}^{*}}\left({\mathrm{r}}_{\langle \mathrm{E}\rangle ,\mathrm{m}\mathrm{i}\mathrm{n}}\right)$	${\langle {\mathrm{E}}_{\mathrm{i}\mathrm{j}}\rangle }^{\mathrm{m}\mathrm{i}\mathrm{n}}$	${\mathrm{E}}_{\mathrm{i}\mathrm{j}}^{{\mathrm{C}}^{*}}\left({\mathrm{r}}_{\langle \mathrm{E}\rangle ,\mathrm{m}\mathrm{i}\mathrm{n}}\right)$	${\langle {\mathrm{E}}_{\mathrm{i}\mathrm{j}}\rangle }^{\mathrm{m}\mathrm{i}\mathrm{n}}$	${\mathrm{E}}_{\mathrm{i}\mathrm{j}}^{{\mathrm{C}}^{*}}\left({\mathrm{r}}_{\langle \mathrm{E}\rangle ,\mathrm{m}\mathrm{i}\mathrm{n}}\right)$	${\mathrm{E}}_{\mathrm{i}\mathrm{j}}^{\mathrm{m}\mathrm{i}\mathrm{n}}$
Et-Et	−0.62	−1.16	−0.62	−1.16	−0.62	−1.16	−1.16
EtOH-Et	−0.75	−1.55	−0.74	−1.45	−0.74	−1.48	−1.52
EtOH-EtOH	−4.75	−6.60	−5.07	−6.87	−4.75	−6.75	−7.30
H2O-Et	−0.31	−0.99	−0.26	−0.79	−0.25	−0.79	−0.81
H2O-EtOH	−7.61	−9.34	−6.48	−8.37	−5.49	−7.57	−10.93
H2O−H2O	−7.85	−9.41	−4.82	−6.54	−4.81	−6.54	−7.27
Me-Et	−0.43	−0.77	−0.43	−0.77	−0.42	−0.77	−0.78
Me-EtOH	−0.51	−0.98	−0.51	−0.95	−0.50	−0.96	−0.98
Me-H2O	−0.23	−0.60	−0.23	−0.50	−0.23	−0.50	−0.52
Me-Me	−0.33	−0.52	−0.33	−0.52	−0.33	−0.52	−0.52
Me2O-Et	−0.79	−1.43	−0.80	−1.40	−0.80	−1.40	−1.44
Me2O-EtOH	−2.95	−5.34	−3.07	−5.31	−3.09	−5.23	−5.58
Me2O-H2O	−4.26	−5.54	−3.15	−4.39	−3.14	−4.38	−4.50
Me2O-Me	−0.55	−0.99	−0.55	−0.99	−0.54	−0.99	−1.00
Me2O-Me2O	−1.61	−2.50	−1.51	−2.39	−1.51	−2.39	−2.40
MeOH-Et	−0.61	−1.50	−0.59	−1.41	−0.59	−1.39	−1.44
MeOH-EtOH	−5.01	−6.62	−5.06	−6.58	−5.09	−6.71	−7.19
MeOH-H2O	−7.31	−8.85	−6.40	−8.22	−6.37	−8.00	−10.85
MeOH-Me	−0.40	−0.94	−0.40	−0.91	−0.40	−0.90	−0.93
MeOH-Me2O	−3.69	−5.35	−3.70	−5.26	−3.65	−5.21	−5.48
MeOH-MeOH	−4.30	−6.21	−4.47	−6.20	−4.44	−6.20	−6.57
HAc-HAc	−11.21	−12.05	−11.90	−12.67	−11.41	−12.27	−13.60

“New variant (original)” uses the same non-bonding parameters as the original calculation pipeline in [3]. “New variant (LigParGen)” uses the non-bonding parameters determined by LigParGen. *H*_*2O*_ Water, *Me* Methane, *MeOH* Methanol, *Me2O* Dimethyl ether, *Et* Ethane, *EtOH* Ethanol, *HAc* Acetic acid

The neglect of the final dimer geometry optimization to obtain ${E}_{ij}^{min}$ may cause significant deviations for larger monomer molecules, e.g., highly polar molecules with multiple polar atoms which form strong, highly directional electrostatic interactions, where small spatial misalignments could cause comparatively large artificial energy spikes, or molecules with a high degree of conformational freedom. The calculation pipeline automatically generates two diagnostic plots for each calculated dimer interaction in its specific output directory: These comprise detailed intermolecular energy-distance plots where unexpected fluctuations, artificial discontinuities, or incomplete convergence can be detected. The sketched unwanted deviations can be effectively mitigated by systematically increasing the configuration sampling size (by increasing ${N}_{sphere}$ and ${N}_{rot}$ correspondingly) to take more configurations into account.

Compared to the established calculation pipeline in [3], the exclusive in-memory calculations with comparable calculation results lead to a performance boost of more than two orders of magnitude–reducing computational “weeks and months” of the established approach to a mere “hours or days” for the new simplified in-memory variant. A direct comparison on an AMD Threadripper Pro 5995WX workstation with 64 calculation threads for calculating the mutual interaction energies of a set of 5 monomer molecules (H2O, Et, EtOH, Me, and Me2O, comprising 15 unique dimer interactions and 144 × 144x16 dimer configurations for each distance, compare Fig. 1) yielded a computation time of 1080.0 s (18 min) until the original file-bound and Tinker-dependent pipeline (version 1.0.3) had completed the task, whilst the new in-memory variant (version 1.0.6) required only 6.1 s for the identical calculations – resulting in a speedup factor of 177 and illustrating the leap in performance achieved by eliminating disk I/O operations and external process overhead. Within a given time, the speed-up enables a significant increase of the number of calculated spatial dimer configurations to improve averages of non-bonded intermolecular interactions at different monomer distances as well as a considerably increased size of the monomer molecule set for calculating mutual interaction energies. While the established pipeline calculates with 144 × 144x16 (= 331,776) dimer configurations for each single average at each distance by default, the new variant routinely uses 1000 × 1000x60 (= 60 million) configurations. As a rule of thumb, the full calculation of a single dimer interaction energy scheme for obtaining ${r}_{\langle E\rangle ,min}$ and corresponding ${\langle {E}_{ij}\rangle }^{min}$ (see Fig. 1 and description above) takes less than 1 min with these 1000 × 1000x60 (60 million) dimer configurations using an AMD Threadripper 5995WX workstation processor with 64 calculation threads.

## Data Availability

All data supporting the findings of this study are available within the paper.

## Abbreviations

BOSS: Biochemical and Organic Simulation System
CPU: Central Processing Unit
DPD: Dissipative Particle Dynamics
LigParGen: Ligand Parameter Generator
OPLS-AA: Optimized Potentials for Liquid Simulations-All-Atom
